# Prediction of outcome of non-small cell lung cancer patients treated with chemotherapy and bortezomib by time-course MALDI-TOF-MS serum peptide profiling

**DOI:** 10.1186/1477-5956-7-34

**Published:** 2009-09-03

**Authors:** Johannes Voortman, Thang V Pham, Jaco C Knol, Giuseppe Giaccone, Connie R Jimenez

**Affiliations:** 1 OncoProteomics Laboratory, Dept Medical Oncology, VUmc-Cancer Center Amsterdam, VU University Medical Center, Amsterdam, the Netherlands; 2 Medical Oncology Branch, National Cancer Institute, National Institutes of Health, Bethesda, MD, USA

## Abstract

**Background:**

Only a minority of patients with advanced non-small cell lung cancer (NSCLC) benefit from chemotherapy. Serum peptide profiling of NSCLC patients was performed to investigate patterns associated with treatment outcome.

Using magnetic bead-assisted serum peptide capture coupled to matrix-assisted laser desorption/ionization time-of-flight mass spectrometry (MALDI-TOF-MS), serum peptide mass profiles of 27 NSCLC patients treated with cisplatin-gemcitabine chemotherapy and bortezomib were obtained. Support vector machine-based algorithms to predict clinical outcome were established based on differential pre-treatment peptide profiles and dynamic changes in peptide abundance during treatment.

**Results:**

A 6-peptide ion signature distinguished with 82% accuracy, sensitivity and specificity patients with a relatively short vs. long progression-free survival (PFS) upon treatment. Prediction of long PFS was associated with longer overall survival. Inclusion of 7 peptide ions showing differential changes in abundance during treatment led to a 13-peptide ion signature with 86% accuracy at 100% sensitivity and 73% specificity. A 5-peptide ion signature could separate patients with a partial response vs. non-responders with 89% accuracy at 100% sensitivity and 83% specificity. Differential peptide profiles were also found when comparing the NSCLC serum profiles to those from cancer-free control subjects.

**Conclusion:**

This study shows that serum peptidome profiling using MALDI-TOF-MS coupled to pattern diagnostics may aid in prediction of treatment outcome of advanced NSCLC patients treated with chemotherapy.

## Background

Lung cancer is the most common cause of cancer-related death with an overall 5-year survival rate of 16% [1]. Non-small cell lung cancer (NSCLC) accounts for approximately 85% of lung carcinomas and is frequently diagnosed in an advanced stage [2]. First-line treatment of advanced NSCLC typically consists of platinum-based chemotherapy [3,4]. Only a minority of patients respond to this treatment, at the cost of substantial toxicity for all treated patients [5]. There is an urgent need for methods enabling outcome prediction in order to select patients likely to benefit from treatment.

The serum peptidome, that comprises peptides and proteins with a molecular weight of less than 10 kDa, represents a dynamic reflection of tissue function in health and disease [6]. Mass spectrometry (MS) is increasingly used to profile the serum peptidome [7-9]. Peaks in the serum peptide spectra correspond to peptide ions, with the amplitude of the peaks indicative of relative abundance [10]. Magnetic bead-assisted serum peptide capture coupled to matrix assisted laser desorption/ionization time-of-flight MS (MALDI-TOF-MS) is a serum peptide profiling strategy gaining in popularity compared to surface-enhanced laser desorption/ionization (SELDI)-based platforms due to superior resolution of MALDI instruments, the possibility to obtain structural (MS/MS) information of signature peptides and superior binding capacity of the magnetic beads compared to a flat SELDI-chip surface [11].

Here we report on a MALDI-TOF-MS dataset of serum samples from advanced NSCLC patients, who were treated with first-line chemotherapy, consisting of cisplatin and gemcitabine, as well as bortezomib, in a previously reported prospective clinical trial [12]. The efficacy of cisplatin-gemcitabine alone is limited, a partial tumor response being achieved in about one third of NSCLC patients and with a median progression free survival of four to five months [13]. Preclinical as well as initial clinical studies suggested combining cisplatin-gemcitabine with proteasome inhibitor bortezomib might enhance efficacy [14-17].

We hypothesized that specific serum peptidome patterns could predict clinical outcome of patients who underwent chemotherapy-based treatment. For this purpose, data analyses of serum peptide profiles were conducted. Our primary aim was to establish serum peptide signatures that could predict positive or negative clinical outcome upon treatment. Clinical endpoints used to establish these signatures were response to treatment according to the Response Evaluation Criteria in Solid Tumors (RECIST) criteria, as well as progression-free survival duration of treated patients. Furthermore, in a secondary analysis, we compared the serum peptide profiles of treated patients with those obtained from cancer-free control subjects in an initial attempt to establish cancer-specific serum peptide patterns.

## Results

### Pre-treatment serum peptide patterns of NSCLC patients

First, pre-treatment serum spectra of 27 NSCLC patients were determined. See Table 1 for patient characteristics. Six hundred eighty-two peaks could be distinguished. The intra-run and inter-run coefficients of variance were 16% and 18%, respectively.

**Table 1:** Patient characteristics

	**NSCLC patients** **(n = 27)**
*Age, years*	
Median	53
Range	35-67
*Sex, n (%)*	
Male	15 (55.6)
Female	12 (44.4)
*Stage, n (%)*	
IIIB	5 (18.5)
IV	22 (81.5)
*Histology, n (%)*	
Squamous cell carcinoma	6 (22.2)
Adenocarcinoma	12 (44.4)
Adenosquamous cell carcinoma	2 (7.4)
Undifferentiated non-small cell carcinoma	7 (25.9)
*Karnofski performance status, n (%)*	
70	4 (14.8)
80	10 (37.0)
90	8 (29.6)
100	5 (18.5)
*Smoking history, n (%)*	
No	1 (3.7)
Current or former	26 (96.3)
*RECIST, n (%)*	
Partial response	9 (33.3)
Stable disease	15 (55.6)
Progressive disease	3 (11.1)
*Survival, days*	
Median progression-free survival	152
Median overall survival	315

### Time-course analysis

**Figure 1:**
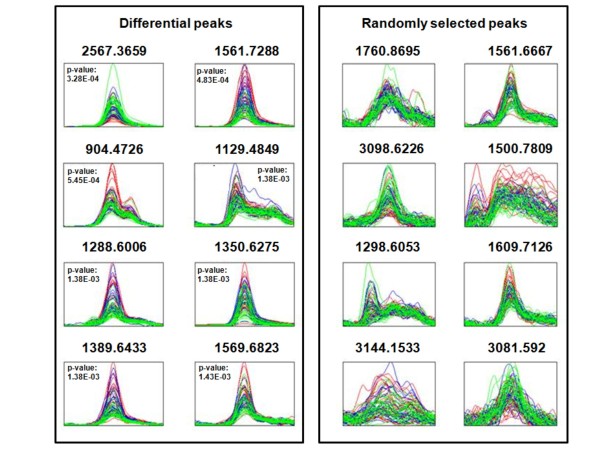
**Comparison serum profiles time course**. Top 8 differential peaks that exhibit changes over the three time points: pre-treatment (PreTx) (red), after two cycles of treatment (Post-2) (blue), and at end of treatment (EOT) (green). Eight peaks randomly selected out of the remaining 638 peaks. Each overlay contains spectra with normalized intensities.

We first looked for peptides that exhibited significant changes in intensity level in three time points: (1) pre-treatment (preTx), (2) after two cycles of treatment (Post-2), and (3) at the end of treatment (EOT). Forty-four peaks were determined as significant (Table 2). The spectra overlay of the top 8 peaks is illustrated in Figure 1. Note that for example the peak at *m/z *2567.3659 has a higher intensity at end of treatment (green) compared to pre-treatment (red) and the peak at *m/z *1561.7288 has a lower intensity at end of treatment compared to pre-treatment.

**Table 2:** Comparison serum profiles time-course

***m/z***	**p-value**	**peptide ID**
904.4726	0.00054514	BK

1129.4849	0.0013845	FPB

1183.5995	0.0094174	

1201.5663	0.0045301	

1263.5958	0.0024011	FPA

1288.6006	0.0013845	

1347.5297	0.047967	

1350.6275	0.0013845	FPA

1389.6433	0.0013845	

1402.671	0.0089624	

1418.539	0.026155	

1432.644	0.039554	

1440.5407	0.011867	

1445.5828	0.0032159	

1447.6855	0.005355	

1450.4893	0.024048	

1456.5093	0.039554	

1458.4946	0.020271	

1460.6257	0.0022586	

1477.6539	0.010181	

1479.6646	0.005355	

1487.6226	0.0017206	

1491.6634	0.0074232	

1501.748	0.011	

1503.5994	0.0087051	

1507.6643	0.0024697	

1536.6906	0.0087051	FPA

1552.669	0.003822	Glu-FBP

1561.7288	0.00048292	

1569.6823	0.0014303	

1573.7005	0.0022586	

1616.6366	0.013817	P-FPA

1630.6679	0.0022586	

1670.5947	0.011876	

1690.9254	0.03325	C3f

1777.966	0.0087051	C3f

1865.0022	0.011	C3f

2494.1536	0.030814	

2567.3659	0.00032806	seAlb

2602.3048	0.019183	CF XIIIA

2743.4663	0.005355	

2747.4349	0.0014303	

2789.0914	0.037043	

3156.6207	0.020803	ITIH4

In NSCLC patients, 44 peaks were significantly differential over the three time points: pre-treatment, after two cycles of treatment and end of treatment. Ordered by *m/z *value. Abbreviations: BK: bradykinin; FBP: fibrinopeptide B; FPA: fibrinopeptide A; Glu-FPB: Glu-1-fibrinopeptide B; P-FPA: phospho-fibrinopeptide A; C3f: complement C3 f; seAlb: serum albumen; CFXIIIA: coagulation factor XIIIA; ITIH4: inter-alpha-trypsin inhibitor heavy chain H4.

### Analysis of the clinical outcome: progression-free survival

The patients were divided into two subsets according to PFS duration. Of the 27 NSCLC patients, 11 patients with the shortest PFS were nominated as "short PFS" group (PFS ≤ 127 days), 11 patients with the longest PFS were nominated as "long PFS" group (PFS ≥ 178 days). Four patients with PFS duration around the median PFS (152 days) were excluded from the analysis. A fifth patient was excluded who had a partial response but died 36 days after start of treatment due to a cause not likely due to tumor progression [12].

**Figure 2:**
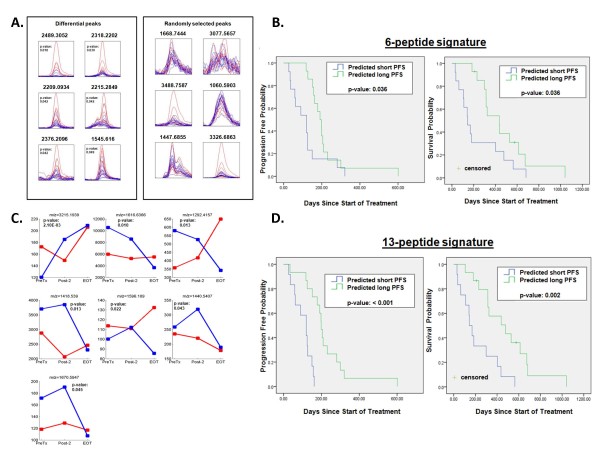
**Comparison serum profiles short PFS vs. long PFS**. **A**, six peaks are differential between the two groups: short PFS (red) and long PFS (blue). Six randomly selected peaks. Each overlay contains spectra with normalized intensities. **B**, Kaplan-Meier (1) time to progression and (2) overall survival curve in NSCLC patients, by prediction of short or long PFS using the 6-peptide signature (intent-to-treat population, n = 27). **C**, median of the seven differential peaks comparing short PFS (red) and long PFS (blue) over the three time points: pre-treatment (PreTx), after two cycles of treatment (Post-2), and at end of treatment (EOT) (Y-axis: intensity).**D**, Kaplan-Meier (1) time to progression and (2) overall survival curve in NSCLC patients, by prediction of short or long PFS using the 13-peptide signature (intent-to-treat population, n = 27).

Six differentially expressed peptides between the two groups were detected (see Figure 2A and Table 3). Median intensity of all six peptides was higher in the "short PFS" group compared to the "long PFS" group. This 6-peptide signature was used to retrospectively divide the total study population (n = 27) into a predicted "short PFS" group and a predicted "long PFS" group. Median PFS was significantly shorter at 120 days (95% CI 54-186 days) in patients predicted to have a short PFS vs. 191 days (95% CI 154-228 days) in patients predicted to have a long PFS (p-value: 0.036). Median overall survival (OS) of patients predicted to have short PFS vs. long PFS was 144 days (95% CI 75-213 days) vs. 436 days (95% CI 292-580 days) (p-value: 0.036) (Figure 2B). For the OS analyses, two patients were censored at last known date to be alive.

**Table 3:** Comparison serum profiles short PFS vs. long PFS

***m/z***	**p-value**
2489.3052	0.018082

2318.2202	0.030239

2209.0934	0.041789**

2215.2849	0.041789

2376.2096	0.041789

1545.616	0.048844

Six peaks were significantly differential comparing NSCLC patients with short progression-free survival and long progression-free survival. All peaks were up-regulated in the group with short progression-free survival. None of the six peaks were positively identified. Ordered by p-value. ** identified by Tempst *et al. *as high-molecular-weight kininogen [19].

When considering also Post-2 and EOT samples, we found seven differential peptides distinguishing the clinical groups (see Figure 2C and Table 4). These peptides do not overlap with peptides from the pre-treatment signature. Using the combined 13-peptide signature to classify the total study population, median PFS was significantly shorter at 120 days (95% CI 70-170 days) in patients predicted to have a short PFS vs. 199 days (95% CI 181-217 days) in patients predicted to have a long PFS (p-value: < 0.001). Median overall survival of patients predicted to have short vs. long PFS was 144 days (95% CI 96-192 days) vs. 478 days (95% CI 203-753 days) (p-value: 0.002) (Figure 2D).

**Table 4:** Comparison time-course serum profiles short PFS vs. long PFS

***m/z***	**p-value**	**peptide ID**
3215.1939	0.0021001	

1616.6366	0.010132	P-FPA

1292.4157	0.013272	

1418.539	0.013272	

1596.189	0.022018	

1440.5407	0.043474	

1670.5947	0.044759	

Seven peaks were significantly differential comparing NSCLC patients with short progression-free survival and long progression-free survival, using three time points: pre-treatment, after two cycles of treatment and end of treatment. Ordered by p-value.

Abbreviation: P-FPA: phospho-fibrinopeptide A.

Finally, we carried out classification analysis using support vector machine. Using all 682 peptides, the LOOCV accuracy was very poor, at about 50%. When the six differential pre-treatment peptides were used, the LOOCV accuracy was 82% with both 82% sensitivity and 82% specificity. Selecting six different peptides randomly resulted in an average accuracy of 68% (10 runs). The LOOCV prediction accuracy improved when we used also the seven peptides that changed differently in intensity level over the three time points. Here, using the 13 peptides we could separate the two groups with a LOOCV accuracy of 86% at 100% sensitivity and 73% specificity (the average accuracy over 10 runs with random selection of 13 peptides was 71%).

### Analysis of the clinical outcome: tumour response

To identify a signature associated with tumour response, we divided the patients into three groups according to tumour response following treatment: (1) partial response (PR), (2) stable disease (SD), (3) progressive disease (PD), as defined by RECIST [18]. Because there were only three patients with progressive disease, we created two groups: PR and SD/PD combined ("no PR"). The first group had 9 patients, and the second group consisted of 18 patients.

**Figure 3:**
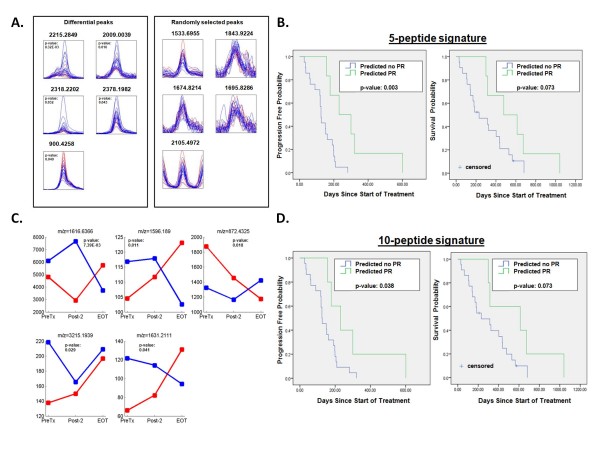
**Comparison serum profiles PR vs. no PR**. **A**, five differential peaks in the comparison between PR (red) versus PD+SD (blue) using pre-treatment samples. Five randomly selected in the same comparison. Each overlay contains spectra with normalized intensities. **B**, Kaplan-Meier (1) time to progression and (2) overall survival curve in NSCLC patients, by prediction of PR or no PR, using the 5-peptide signature (intent-to-treat population, n = 27). **C**, median of the five differential peaks in the comparison between PR (red) versus PD+SD (blue) using three time points: pre-treatment (PreTx), after two cycles of treatment (Post-2), and at end of treatment (EOT). **D**, Kaplan-Meier (1) time to progression and (2) overall survival curve in NSCLC patients, by prediction of PR or no PR, using the 10-peptide signature (intent-to-treat population, n = 27).

Comparing pre-treatment samples, we detected five differential peaks (see Figure 3A and Table 5). Of the five peptides, two were also present in the list of differential peaks comparing short PFS and long PFS (*m/z *= 2215.2849 and 2318.2202). The 5-peptide signature was used to retrospectively divide the total patient population in a PR vs. no PR group (n = 27). In the patient group classified as no PR, median PFS was significantly shorter at 125 days (95% CI 115-135 days) vs. 231 days (95% CI 85-377 days) in the group classified as PR (p-value: 0.003). Median overall survival of patients predicted to have no PR was shorter, but not significantly, at 231 days (95% CI 41-421 days) vs. 478 days (95% CI 120-836 days) for patients predicted to have a PR (p-value: 0.073) (Figure 3B).

**Table 5:** Comparison serum profiles PR vs. no PR

***m/z***	**p-value**
2215.2849	0.0083178

2009.0039	0.015663

2318.2202	0.032445

2378.1982	0.042583

900.4258	0.048584

Five peaks were significantly differential comparing NSCLC patients with a partial response versus patients with stable or progressive disease. None of the 5 peptides were positively identified. Ordered by p-value.

Next we included also the Post-2 and EOT samples, resulting in five additional differential peptides (see Figure 3C and Table 6). Again, there was no overlap between these five peptides and the peptides found when using the pre-treatment samples only. However, of these five peptides, three peptides were also in the list of significant peptides when comparing short PFS and long PFS over the three time points (*m/z *= 1596.189, 1616.637 and 3215.194). The 10-peptide signature was used to retrospectively divide the total patient population in PR vs. no PR (n = 27). In the patient group classified as no PR, median PFS was 125 days (95% CI 91-159 days) vs. 231 days (95% CI 117-345 days) in the group classified as PR (p-value 0.038). Median overall survival of patients predicted to have no PR was shorter, but not significantly, at 231 days (95% CI 36-426 days) vs. 613 days (95% CI 0-1253 days) for patients predicted to have a PR (p-value: 0.077) (Figure 3D).

**Table 6:** Comparison time-course serum profiles PR vs. no PR

***m/z***	**p-value**	**peptide ID**
1616.6366	0.007392	P-FPA

1596.189	0.011159	

872.4325	0.018308	

3215.1939	0.028873	

1631.2111	0.040931	

Five peaks were significantly differential comparing NSCLC patients with a partial response versus patients with stable or progressive disease, using three time points: pre-treatment, after two cycles of treatment and end of treatment. Ordered by p-value.

Abbreviation: P-FPA: phospho-fibrinopeptide A

**Figure 4:**
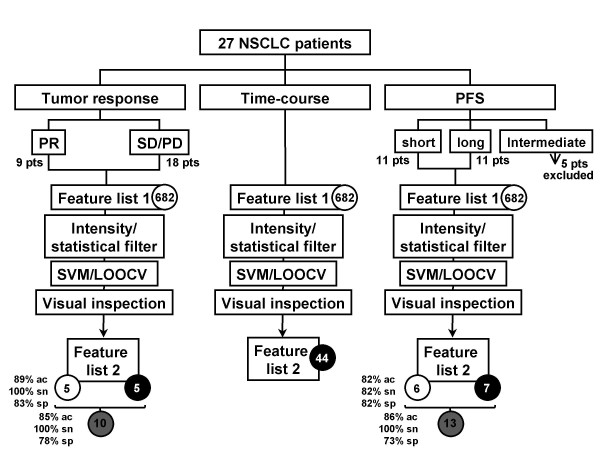
**Study flowchart**. Serum profiling was performed in 27 NSCLC patients. "Feature list 1" represents total number of features before intensity and statistical filters. For each comparison, "Feature list 2" represents the number of differential peptide ions comparing pre-treatment sera (white filled circle), time-course sera (black filled circle) and pre-treatment plus time-course combined (grey filled circle). Accuracy (ac), sensitivity (sn) and specificity (sp) of algorithms are indicated. SVM: support vector machine; LOOCV: leave-one-out-cross validation; pts: patients.

Classification analysis was performed using support vector machine with different combinations of features. The LOOCV accuracy was again poor when using all 682 peptides, at about 67%. When the five differential pre-treatment peptides were used, the LOOCV accuracy was 89% at 100% sensitivity and 83% specificity. The average accuracy over 10 runs was 74% using random feature selection for five peptides. The LOOCV prediction accuracy was reduced to 85%, at 100% sensitivity and 78% specificity, when we used also the five peptides that changed differently in intensity level over the three time points (Figure 4 provides an overview of the study and study results).

### Peptide pattern discriminating NSCLC patients from cancer-free controls

**Figure 5:**
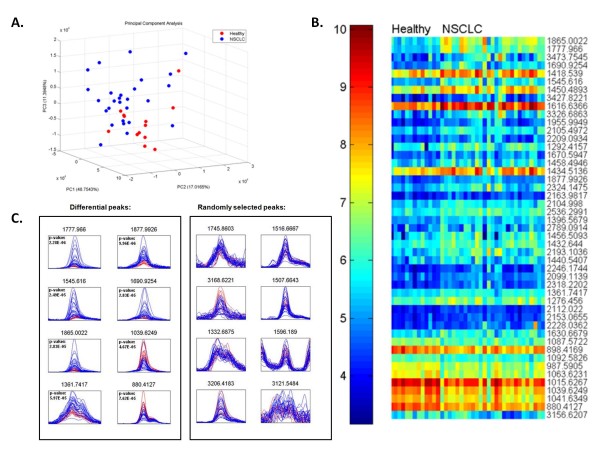
**Comparison serum profiles NSCLC vs. cancer-free controls**. **A**, Principle Component Analysis (PCA) NSCLC vs. cancer-free control comparison. **B**, heat map of the 47 differential peaks. The peaks are ordered by median fold change between the two groups. **C**, spectra overlay of the 8 most differential peaks in the healthy (red) versus NSCLC (blue) comparison. Spectra overlay of the 8 peaks randomly selected out of the remaining 635 peptides.

Finally, in an exploratory additional analysis, we compared the serum peptide spectra of 13 cancer-free control subjects (median age: 38 years old; range: 27-58 years old) and the pre-treatment serum spectra of the 27 NSCLC patients included in this study. We performed a principal component analysis (PCA) analysis of the 40 profiles using all 682 peptides, see Figure 5A. While there is overlap between the two groups in the three dimensional plot, the healthy profiles (in red) are clustered at the bottom right region. Furthermore, we observed no indication of outliers in the dataset. Next we performed a supervised analysis to identify peptides that were significantly differential in intensity between the two groups. For this purpose, the Mann-Whitney U test was carried out on each of the 682 peptides using all profiles. The peptides were selected based on the criteria outlined in Methods, resulting in 47 peptides. A heat map of the intensities of the 47 peptides is shown in Figure 5B (see also Table 7). Figure 5C shows the spectra overlay of the top 8 most discriminating peaks, all of which have a p-value < 0.0001. Note that for example the peak at m/z 1777.966 has a higher intensity in NSCLC patients (blue) compared cancer-free controls (red) and the peak at m/z 1039.6249 has a lower intensity in NSCLC patients. We carried out classification analysis using support vector machine. A grid search for parameters was employed to find the best model according to LOOCV. Using all 682 peptides, an LOOCV accuracy of 93% was achieved. When the 47 peptides selected by the Mann-Whitney U test were used, the LOOCV accuracy was 98% with 100% sensitivity and 96% specificity. To substantiate the result, we compared it to a random selection of peptides. Using the same model selection mechanism for support vector machine with 47 different peptides randomly selected the average accuracy over 10 runs was 90%.

**Table 7:** Comparison serum profiles NSCLC vs. cancer-free controls

***m/z***	**p-value**	**peptide ID**
1777.966	2.18E-06	C3f

1877.9926	9.96E-06	

1545.616	2.49E-05	

1690.9254	2.83E-05	C3f

1865.0022	2.83E-05	C3f

1039.6249	4.67E-05	

1361.7417	5.97E-05	

880.4127	7.62E-05	

1041.6349	0.00010908	

1955.9949	0.00013801	

1015.6267	0.00017407	

1087.5722	0.00017407	

1063.6231	0.00034269	

1092.5826	0.00034269	

2318.2202	0.00034269	

2536.2991	0.00034269	

2105.4972	0.00047573	

1396.5679	0.0005897	

2112.022	0.00072868	

987.5905	0.0013492	

1616.6366	0.0016465	P-FPA

2324.1475	0.0018167	C3 beta

2104.998	0.0022066	

2153.0655	0.0024291	

1450.4893	0.0026719	

2193.1036	0.0032253	C3 beta

898.4169	0.0035395	

3427.8221	0.0035395	

2163.9817	0.0046562	

1670.5947	0.0050941	

2099.1139	0.0072417	

1456.5093	0.0078632	

1418.539	0.0078923	

1630.6679	0.0078923	

2228.0362	0.0078923	

2209.0934	0.0085948	**

3326.6863	0.0085948	Hb alpha

1434.5136	0.01017	

3473.7545	0.01017	Hb alpha

1432.644	0.014108	

1292.4157	0.01933	

1440.5407	0.024299	

2246.1744	0.030331	

2789.0914	0.032609	

3156.6207	0.035032	ITIH4

1276.456	0.037607	

1458.4946	0.040342	

47 peaks were significantly differential comparing NSCLC patients and healthy volunteers. Ordered by p-value. ** identified by Tempst *et al. *as high-molecular-weight kininogen [19]. Abbreviations: P-FPA: phospho-fibrinopetide A; C3 beta/f: complement C3 beta/f; Hb alpha: hemoglobin alpha; seAlb: serum albumen; ITIH4: inter-alpha-trypsin inhibitor heavy chain H4

In this secondary analysis, control subjects were unmatched for age and gender. We therefore also considered peptides that express differently in the two gender groups. For this comparison, four peptides were differential according to our criteria described above (*m/z *= 1458.495, 3215.194, 2602.305, and 2789.091). Of these four peptides, two are present in the list of 47 differential peptides in the healthy versus NSCLC comparison. Ignoring these two peptides, the signature composed of the remaining 45 peptides yielded the same accuracy, sensitivity and specificity as that of the 47-peptide signature. Literature supports that serum peptidome patterns that distinguished advanced cases of cancer from cancer-free controls were unbiased by gender and age, except for the fact that healthy subjects under 35 years could be distinguished with approximately 70% accuracy [19]. All participating patients in our study were 35 years or older. However, in the cancer-free control group, 4 individuals were younger than 35 years and 9 individuals older than 35 years. Comparing these two groups, two peaks met the criteria for differential (m/z = 1020.5133 and 855.0387). These two peaks did not feature in the classifying signature between the NSCLC patients and the cancer-free controls.

### Peptide identification

**Figure 6:**
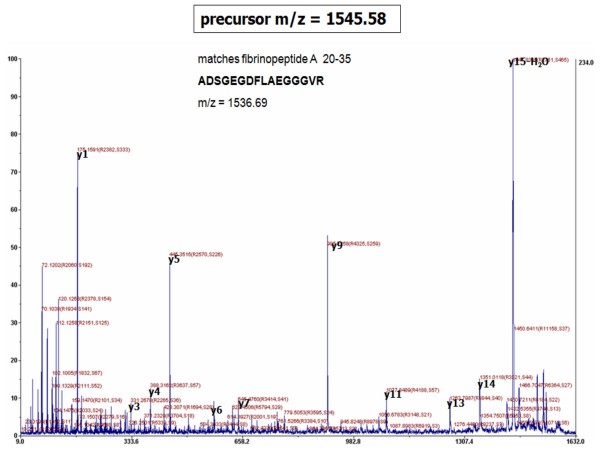
**Tandem MS-based peptide identification**. As an example of tandem-MS generated spectra, the annotated MS/MS spectrum of precursor mass 1545.58 is displayed.

For structural identification of signature peptides by MS/MS, we performed an additional peptide capture on another aliquot of the sera used for profiling. Sera with highest intensity levels of signature ions were selected for MS/MS. For each eluate, a series of four spots was applied to a MALDI target plate, and candidate peaks were subjected to MS/MS in the sample spot(s) associated with the highest intensity for the pertinent peak. Seventeen peptides were positively identified by MALDI-TOF/TOF-based MS/MS analysis, see Table 8 as well as Tables 2, 4 and 6. See Figure 6 for an example of an annotated MS/MS spectrum. In agreement with results by Villanueva *et al. *in other tumor types, the serum peptide signatures mainly consisted of small sets of overlapping sequences, truncated in both ends in a ladder-like fashion. See Table 8 for truncation ladder examples of Fibrinopeptide alpha, Complement C3f, Complement C3 beta and Hemoglobin alpha.

**Table 8:** MS/MS identification results

**Peptide ID**	***m/z***	**Peptide residue location**	**Peptide amino acid sequence**	**Signature(s)**
**BK**	904.4676	381-388	RPPGFSPF	Time course (44)
**P-FPA**	1616,6366	20-35	ADS*GEGDFLAEGGGVR	Time course (44); PFS (13); Response (10); NSCLC vs. Healthy (47)
**FPA**	1536,6906	20-35	ADSGEGDFLAEGGGVR	Time course (44)
**FPA**	1350,6275	22-35	SGEGDFLAEGGGVR	Time course (44)
**FPA**	1263,5958	23-35	GEGDFLAEGGGVR	Time course (44)
**Glu-FPB**	1552.669	31-44	EGVNDNEEGFFSA	Time course (44)
**FPB**	1129.4849	34-43	NDNEEGFFSAR	Time course (44)
**C3f**	1865,0022	1304-1319	SSKITHRIHWESASLL	Time course (44)
**C3f**	1777,966	1305-1319	SKITHRIHWESASLL	Time course (44)
**C3f**	1690,9254	1306-1319	KITHRIHWESASLL	Time course (44)
**C3 beta**	2324,1475	23-42	SPMYSIITPNILRLESEETM	NSCLC vs. Healthy (47)
**C3 beta**	2193,1036	23-41	SPMYSIITPNILRLESEET	NSCLC vs. Healthy (47)
**Hbα**	3473,7545	2-34	VLSPADKTNVKAAWGKVGAHAGEYGAEALERMF	NSCLC vs. Healthy (47)
**Hbα**	3326,6863	2-33	VLSPADKTNVKAAWGKVGAHAGEYGAEALERM	NSCLC vs. Healthy (47)
**seAlb**	2567,3659	27-48	HKSEVAHRFKDLGEENFKALVL	Time course (44)
**ITIH4**	3156,6207	617-644	NVHSGSTFFKYYLQGAKIPKPEASFSPR	Time course (44); NSCLC vs. Healthy (47)
**CFXIII A**	2602,3048	43-67	AVPPNNSNAAEDDLPTVELQGVVPR	Time course (44)

Peptides were identified from the 44-peptide ion time course signature ("time course (44)"), the 13-peptide ion "long PFS" vs. "short PFS" signature ("PFS (13)"), the 10-peptide ion "PR" vs. "no PR" signature ("Response (10)") and the 47-peptide ion NSCLC vs. Healthy comparison ("NSCLC vs. Healthy (47)"). Abbreviations: FPA: fibrinopeptide A; P-FPA: phospho-fibrinopeptide A (phosphorylation site indicated by *); FBP: fibrinopeptide B; Glu-FBP: Glu-1-Fibrinopeptide B; C3f: complement C3f; C3b: complement C3beta; Hb alpha: hemoglobin alpha; seAlb: serum albumen; ITIH4: inter-alpha-trypsin inhibitor heavy chain H4; CFXIIIA: coagulation factor XIIIA.

## Discussion

In this study, we investigated the use of serum peptide mass profiling by MALDI-TOF-MS coupled to bioinformatics pattern discovery to predict treatment outcome of advanced NSCLC patients treated with platinum-based therapy. Additionally, peptide patterns found in NSCLC patients were differential from those found in healthy volunteers.

To our knowledge we are the first to report on a serum peptide signature for response and survival prediction in NSCLC patients treated with cisplatin-based chemotherapy. For this study, serum samples were obtained not only pre-treatment, but also during treatment and after completion of treatment, whereas serum proteomics studies typically focus on pre-treatment samples only. In a study by Taguchi *et al.*, a predictive MALDI-TOF-MS-based peptide algorithm for "good" or "poor" clinical outcome upon epidermal growth factor tyrosine kinase inhibitor (EGFR-TKI) therapy was established [20]. However, in this study none of the peptides corresponding to the classifying *m/z *features were identified.

In our study, for the short PFS versus long PFS classification, we achieved 82% accuracy with a 6-peptide-signature. A longer overall survival of the patients classified as "long PFS" and a shorter overall survival of the patients classified as "short PFS" was observed. Interestingly, performance of the signature was improved upon inclusion of 7 differential time-course peptides, the combined 13 peptide-ion signature achieving 86% accuracy. In this regard, it has been previously reported that histological response of locally advanced rectal cancer to radiochemotherapy could be predicted by SELDI-TOF-MS-based profiling and comparison of serum samples collected pre-treatment and during treatment [21].

For partial responders versus non-partial responders we achieved 89% accuracy with a 5-peptide signature. Inclusion of differential time-course peptides did not improve performance of this signature. Longer duration of progression-free survival was strongly associated with tumour response as seven out of eleven patients in our study with long PFS also had a partial tumour response upon treatment, compared to one out of eleven patients in the short PFS group. This suggests that the survival signature is predictive of therapy-outcome rather than prognostic. As the Kaplan-Meier analysis showed, prediction of response using the 5- or 10-peptide signatures was significantly associated with a longer median PFS of those patients, but this did not reach significance for overall survival.

In several MALDI serum peptide profiling studies involving other solid tumour types (bladder, breast, prostate, thyroid) by Villanueva *et al.*, methodologically comparable to our study, the hypothesis was put forward that cancer-type specific changes in exopeptidase activities yield surrogate biomarkers, reflected in the differential abundance of cleavage products of common serum substrate proteins [19,22]. The changes in exopeptidase activity were superimposed on the proteolytic events of the complement degradation and *ex vivo *coagulation pathways and therefore serum-specific. Regulated peptides at *m/z *1690.925, 1777.966 and 1865.002 correspond to those identified in our study at 1690.90, 1777.94 and 1864.95, respectively, previously identified by MALDI-TOF-TOF-based MS/MS analysis as Complement C3f. The peptide at 2209.093 corresponds to the one we detected at 2209.08, previously identified as HMW Kininogen [19].

We show that in NSCLC, the identified differential serum peptides changing in abundance over time and those distinguishing NSCLC patients from cancer-free control subjects consisted of truncated sequence clusters. The identified peptides were derived from naturally occurring serum peptides and protein precursors, and therefore not likely to be tumor-derived, thus supporting the exoprotease hypothesis. For the predictive algorithms, it is important to realize that these might be specific for the treatment combination of bortezomib, cisplatin and gemcitabine and may not apply to other treatment regimens. For this exploratory analysis, we did not perform a power calculation beforehand as we did not know the biological variation in our patient groups, nor the number of peaks we would measure as well as other variables. We only knew the technical variation. It was therefore our approach to collect as many samples as we could accommodate. It is crucial to validate and adjust the established signatures with an independent cohort in a sufficiently powered follow-up study. Additionally, since only one report excludes an age and gender bias for a cancer-specific serum peptide signature, it is advisable to include matched cancer-free control groups for the establishment of cancer-specific peptide patterns.

## Conclusion

Ideally, serum peptide mass profiling can be used to identify the therapeutic agents to which the tumour is sensitive, enabling personalized medicine. The method employed here requires readily accessible, non-invasively obtainable patient samples, is high-throughput and cost-efficient, all together important requirements for a screening platform as well as routine clinical use. The biggest challenge might very well remain lack of reproducibility related to sample collection in the clinic. In particular, maintaining a constant and precise clotting time is often difficult in clinical practice. Potentially, after initial discovery of classifying algorithms, functional proteomics tests will facilitate clinical implementation [23].

## Methods

### Patients and serum preparation

The training set included 27 patients with NSCLC who were treated with chemotherapy and bortezomib as well as 13 healthy volunteers [12]. All patients were treated with cisplatin 70 mg/m^2 ^day 1 and gemcitabine 1,000 mg/m^2 ^days 1 and 8, every 21 days for up to 6 cycles. Fifteen patients were treated with bortezomib on days 1 and 8 of every cycle (ten patients at 1.0 mg/m^2 ^and five patients at 1.3 mg/m^2^). Twelve patients were treated with bortezomib on days 1,4,8 and 11 of every cycle (one patient at 0.7 mg/m^2 ^and eleven patients at 1.0 mg/m^2^). There was no indication of superior clinical activity of any schedule of bortezomib in combination with cisplatin and gemcitabine [12]. Blood samples were obtained in BD Vacutainer glass "red-top" tubes (Becton Dickinson, Franklin Lakes, NJ), allowed to clot for 1 hour, and then centrifuged at 1500 g for 10 minutes. Sera were stored in polypropylene cryovials (Nunc; Roskilde, Denmark) at -80°C. Studies were performed after obtaining patient consent and under protocols approved by the institutional review board.

### Serum sample processing and mass spectrometry

Samples were processed in randomized order, along with control samples to check consistency in each experiment. Magnetic Dynabeads^® ^RPC 18 (Invitrogen, San Diego, CA) were used for serum peptide capture using the KingFischer96 platform, as described previously [11]. Briefly, in a 96-well format, magnetic beads were washed and equilibrated twice in 200 μl 200 mM NaCl/0.1% TFA, transferred to a mix of 20 μl serum sample and 2 volumes of 0.2% n-octyl glucoside/0.5% TFA (premixed for 5 min), incubated for 2 min, washed thrice with 0.1% TFA, and eluted for 2 min with 40 μl 50% acetonitrile.

Processed samples (1.5 μl eluate) were mixed with 2 volumes α-cyano-4-hydroxycinnamic acid matrix (6.2 mg/ml in 56% acetonitrile, 36% methanol; Agilent, Santa Clara, CA) and 0.7 μl of this mix was spotted on a MALDI plate. A 4800 MALDI-TOF/TOF mass spectrometer (Applied Biosystems, Foster City, CA) was used to record with 5000 shots per spectrum (reflectron mode) serum peptide profiles in the mass range of *m/z *800-4000 [11]. Internal calibration was used using a list of exact masses for fibrinogen α/fibrinopeptide A peptides as major components of serum samples. For MS/MS analysis, stepwise attempts of 5000 shots at a time generated spectra for identification by the Mascot search engine or manual identification. For Mascot searches, the SwissProt database (release 54.7) was used at a mass window of 10 ppm for MS and a 1-Da tolerance for MS/MS. Final scores were obtained by narrowing down the window. For manual identification, spectra were compared with theoretical peptide fragments of reported candidate proteins [19]. Fragments having a predicted mass differing less than 10 ppm from the mass of any of the 87 significantly regulated peaks from our profiling study were identified using FindPept [19]. Fragmentation patterns were predicted using MS-Product , requiring that at least 3 prominent peaks in the experimental spectrum should match b or y ions from the theoretical table.

### Signal processing

Spectra were pre-processed using MarkerView, version 1.2 (Applied Biosystems) with a mass tolerance of 200.0 ppm and minimum intensity at 100.0 units. Total signal intensity of all peptide peaks was used for normalization.

### Statistical analysis

Feature selection was performed using the Mann-Whitney U test on each peptide detected in the pre-processing step. We used a common threshold of 5% for the p-value. As p-values were not adjusted for multiple testing, we took additional measures to guard from false discovery. To reduce differences due to noise, each peptide was subjected to intensity filtering, requiring that the median intensity of at least one group must be greater than 80 units and the fold change of the median intensities of the two groups must be greater than 1.5. For time course analysis of the three time points, we treated the problem as three binary comparisons. For each comparison, a paired, two-sided signed rank test was carried out. Each peptide was again subjected to intensity filtering. The results of the three comparisons were merged where the significance level of each peptide was the minimum of the three p-values. Similarly, we analyzed dynamic peptide profiles (time-course), using the group information, in order to identify peptides of which the intensity level changes differently between different clinical groups. Finally, support vector machine with the Gaussian kernel was used to construct classification models. A two dimensional grid search was carried out to set model parameters using the leave-one-out cross-validation (LOOCV) measure. Analogously to Villanueva et al. [19], we used a statistical test for feature selection. This procedure is based on class label, thus bias might be introduced. Nevertheless, this strategy was shown to be effective in previous studies [19,22]. Median duration of progression-free and overall survival was calculated using the Kaplan-Meier method; p-values according to the log-rank test (SPSS Statistics17.0, SPSS Inc., Chicago, IL).

## List of abbreviations

CI: confidence interval; EOT: end of treatment; LOOCV: leave-one-out cross-validation; MALDI-TOF MS: matrix-assisted laser desorption/ionization time-of-flight mass spectrometry; MS: mass spectrometry; NSCLC: non-small cell lung cancer; OS: overall survival; PCA: principal component analysis; PD: progressive disease; PFS: progression-free survival; PR: partial response; PreTx: pre-treatment; RECIST: Response Evaluation Criteria in Solid Tumors; SD: stable disease; SELDI: surface-enhanced laser desorption/ionization.

## Competing interests

The authors declare that they have no competing interests.

## Authors' contributions

JV, TVP and JCK carried out experimental procedures and drafted manuscript. CRJ and GG conceived of the study, and participated in its design and coordination and helped to draft the manuscript. All authors read and approved the final manuscript.
